# A106 THE EPIDEMIOLOGY OF COMPLEX COLONIC POLYPS: A POPULATION BASED STUDY OF THE SOUTHWEST ONTARIO COLONOSCOPY COHORT

**DOI:** 10.1093/jcag/gwab049.105

**Published:** 2022-02-21

**Authors:** A Almudaires, S Alqahtani, V Siebring, C Mcdonald, S Lee, M Sey, B Yan

**Affiliations:** 1 Lawson Health Research Institute, London, ON, Canada; 2 Southwest Regional Cancer Program, London, ON, Canada; 3 Department of Surgery, Western University, London, ON, Canada; 4 Department of Medicine, Western University, London, ON, Canada

## Abstract

**Background:**

Complex polyps are well recognized amongst endoscopists, but its definition varies in the literature and from one endoscopist to another. Despite its clinical importance, the epidemiology of complex polyps is poorly understood.

**Aims:**

To assess the epidemiology of complex polyps on a population level, and in FIT positive individuals.

**Methods:**

The Southwest Ontario Colonoscopy cohort is a prospective database consisting of all adult patients undergoing colonoscopy at 21 hospitals in Southwest Ontario. Data is collected through a mandatory quality assurance form completed by the endoscopist after each procedure. All outpatient adult colonoscopies for any indication were included. Incomplete colonoscopies, repeat procedures, and poor preparation colonoscopies were excluded. A manual review of the colonoscopy report was completed in cases where the description of the complex polyps was missing. The primary outcomes were the prevalence of complex polyps in the cohort, and in FIT positive patients. Secondary outcomes include endoscopic description of the complex polyp, rates of attempted and complete resection, and identification of possible associations between patient and endoscopist factors with complex polyp detection and removal. A multivariate logistic regression model was generated to assess for factors associated with complex polyp detection.

**Results:**

From February 2019 to December 2020, 43389 colonoscopies were included, of which 1459 were for FIT positive patients. 2294 patients had a complex polyp, with a prevalence of 5.3% [95% CI 0.051–0.055], while the prevalence was 17.1% [95% CI 0.152–0.191] in the FIT positive cohort. Compared to average-risk patients undergoing colonoscopy for colon cancer screening, the odds ratio (OR) of detecting a complex polyp in individuals with positive FIT was 4.12 [95% CI 3.42–4.98, p<0.0001]. Among complex polyps,1324 (57.7%) were described as large (>2cm) and 1290 (56%) described as sessile. Of 2294 patients with complex polyps,1992 (86.8%) [95% CI 0.855–0.882] underwent a removal attempt, with successful complete removal as determined by the endoscopist achieved in 1905 patients (95.6%) [95% CI 0.947–0.965]. Compared to gastroenterologists, general surgeons and internists were less likely to detect a complex polyp, OR 0.67 [95% CI 0.61–0.73, p <0.0001] and 0.36 [95% CI 0.20–0.67, p=0.0011] respectively. Trainee involvement was associated with higher rate of complex polyp detection, OR 1.20 [95% CI 1.07–1.35, p=0.0022]. Females were less likely to have a complex polyp compared to males, OR 0.71 [95% CI 0.65–0.77, p<0.0001].

**Conclusions:**

Complex polyps are more prevalent than previously reported in the literature, with a high prevalence among the FIT positive population compared to other indications of colonoscopy.

**Funding Agencies:**

None

